# Structures of GapR reveal a central channel which could accommodate B-DNA

**DOI:** 10.1038/s41598-019-52964-2

**Published:** 2019-11-13

**Authors:** Michael J. Tarry, Christoph Harmel, James A. Taylor, Gregory T. Marczynski, T. Martin Schmeing

**Affiliations:** 1 Department of Biochemistry, McGill University, 3649 Promenade Sir William Osler, Montreal, QC H3G 0B1 Canada; 2 Department of Microbiology and Immunology, McGill University, 3775 University Street, Montreal, QC H3A 2B4 Canada; 3 Present Address: Department of Cellular and Molecular Medicine, University of Ottawa, 451 Smyth 15 Road, Ottawa, ON K1H 8L1 Canada

**Keywords:** X-ray crystallography, DNA replication

## Abstract

GapR is a nucleoid-associated protein required for the cell cycle of *Caulobacter cresentus*. We have determined new crystal structures of GapR to high resolution. As in a recently published structure, a GapR monomer folds into one long N-terminal α helix and two shorter α helices, and assembles into a tetrameric ring with a closed, positively charged, central channel. In contrast to the conclusions drawn from the published structures, we observe that the central channel of the tetramer presented here could freely accommodate B-DNA. Mutation of six conserved lysine residues lining the cavity and electrophoretic mobility gel shift experiments confirmed their role in DNA binding and the channel as the site of DNA binding. Although present in our crystals, DNA could not be observed in the electron density maps, suggesting that DNA binding is non-specific, which could be important for tetramer-ring translocation along the chromosome. In conjunction with previous GapR structures we propose a model for DNA binding and translocation that explains key published observations on GapR and its biological functions.

## Introduction

In prokaryotes, control of DNA replication and related processes for cell division are aided by a family of DNA-binding proteins known as nucleoid-associated proteins (NAPs)^1^. GapR is an essential NAP in *Caulobacter crescentus* involved in DNA replication, chromosome segregation and cell division^2–4^. It has been shown to bind DNA both *in vivo* and *in vitro*^3^. A preference for AT-rich DNA has been reported, though the strength of this preference differs between reports^2,4^. GapR has dynamic chromosome-binding patterns and changing GapR distributions during the cell cycle. In *C. crescentus* “swarmer” cells before chromosome replication, fluorescently tagged GapR shows a concentration gradient across the whole chromosome from high GapR at the origin of replication region to low GapR towards the terminus region^3,4^. Upon starting chromosome replication, a GapR-depleted region of the chromosome is created that expands while tracking the mitotic-like movements of the newly duplicated DNA^3^ and the moving replication forks^4^. Chromatin immunoprecipitation (ChIP) experiments showed that GapR preferentially binds to the 3′ ends of actively transcribed genes, and that blocking transcription with antibiotics quickly (within minutes) dissipates GapR peaks and redistributes GapR towards the 5′ ends of these genes^3,5^. Arias-Cartin *et al*. proposed that replication forks encounter and dislodge tightly bound (presumed static) GapR molecules and that GapR molecules released into the cytoplasm rebind the chromosome at random places^4^.

GapR does not resemble other DNA binding proteins. It is a small protein of ~90 amino acids, with mainly α-helical secondary structure, and until very recently its mode of DNA binding was unknown. Last year, while this work was underway, a highly informative study which included crystal structures of GapR in the presence and absence of DNA was published^5^. In the reported structure of GapR determined in absence of DNA, GapR is seen to be a dimer, with each monomer folded into two extended α helices. In the structure of GapR determined in presence of DNA, GapR is seen to be a tetramer, with the C-terminal α helix of each monomer reorganized into two shorter helices, which allows GapR to encircle the DNA. The DNA in the published structure is 100% AT, over-twisted and slightly narrower than B-form DNA. In the accompanying experiments, GapR is proposed to associate with positively supercoiled chromosomal DNA^5^, which is often found ahead of the replication fork and RNA polymerase^6^. GapR was found to stimulate *in vitro* the topoisomerases gyrase and topo IV, and removing supercoils is required for DNA replication to proceed *in vivo*^5^.

In this paper we present three crystal structures of GapR from *C. crescentus*, crystallised in the presence of double-stranded DNA with a sequence from the origin of chromosome replication. In all three structures, GapR adopts the same overall tetramer ring form as seen by Guo *et al*., albeit with small but important increases in the diameter of the central channel. DNA could not be visualized in any of our three structures, but DNA could be detected in gel electrophoresis, indicating that DNA is present in the crystals. Furthermore, these crystals do not form without DNA in the crystallization protocol. Notably, double stranded B-form DNA can be docked into the channel of our structure without any clashes. We identified residues that our structural analyses suggested should be involved in DNA binding and showed that mutation of these residues abolishes DNA binding while retaining tetramer oligomerization. We propose that our crystals capture a physiologically-relevant GapR structure distinct from and complementary to the physiologically-relevant structures captured by Guo *et al*. Finally, we combine these structural data and present an updated model for DNA binding and translocation which more fully accounts for the observed dynamics of GapR binding.

## Results

### Structure determination of GapR

At the time of undertaking these experiments, no structural information on GapR was known. We set out to crystallize GapR alone and in the presence of DNA to structurally characterize the protein and protein-DNA complex. Initial attempts to crystallize GapR in the absence of added DNA yielded abundant microcrystals. Despite extensive efforts, conditions producing these microcrystals could not be optimized to yield diffraction quality crystals. We next tried crystallizing GapR in the presence of double stranded DNA (dsDNA). For this, we used both full length GapR and GapR_Δ1-11_ (a construct in which we removed the first eleven residues, that were predicted to be disordered) and a 19-bp dsDNA oligomer with a sequence from the *C. crescentus* origin of replication (Table 1), which GapR binds with a nanomolar dissociation constant^3^. GapR-DNA complexes were subjected to sparse matrix crystallization, and multiple crystallization conditions were identified. A single condition was optimized to yield three sets of morphologically distinct diffraction quality crystals, which were used to determine three independent structures of GapR (Table 2).

**Figure 1:**
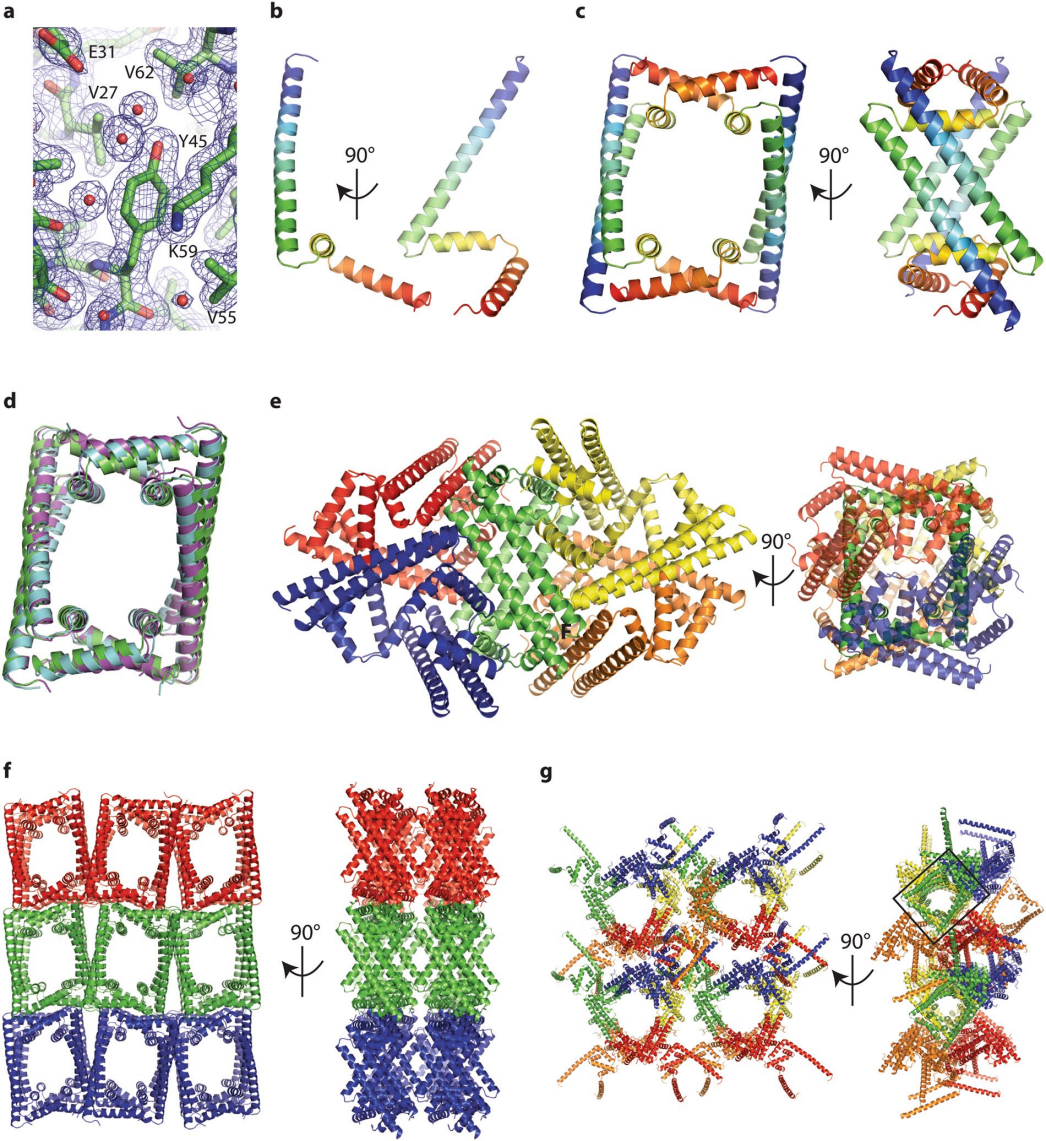
Crystal structures of GapR and packing in the crystal lattice. (**a**) GapR modelled in to a 2F_o_-F_c_ electron density map, shown contoured at 1σ. (**b**) The GapR monomer from the asymmetric unit of the crystals of space group I4_1_32, colored blue through to red from the N to C termini. (**c**) Four symmetry related monomers assemble into the physiologically relevant tetramer. The left side of panel C shows the central channel. (**d**) Overlay of the biological tetramer of GapR from crystals of space group I4_1_32 (green), P222_1_ (cyan) and P4_3_22 (purple) used in this study. (**e**) Packing in crystals of space group I4_1_32, with different tetramers shown in red, blue, green, yellow and orange. The right side of panel e orients the channel of the green tetramer toward to viewer. (**f**) Packing in crystals of space group P222_1_. The left side of panel f orients the channels of all tetramers toward the viewer. (**g**) Packing in crystals of space group P4_3_22. The channel formed by a tetramer is highlighted by a black rectangle in the right side of the panel.

The best diffracting crystals were of full-length GapR plus DNA. A diffraction dataset was collected which included reflections to 1.85 Å resolution and was of the space group I4_1_32. At the time, there were no available structures of GapR, and the protein shared no substantial identity with any other DNA-binding protein structures. Attempts to use DNA as a search model in molecular replacement were unsuccessful. GapR was categorized as containing the domain of unknown function 2312^2,4^ which had previously been subjected to *ab initio* modelling and theoretical screening for nucleic acid binding potential^7^. We submitted the sequence of GapR to the Roβetta server for protein structure prediction^8^, and the top five models produced were used as search models in molecular replacement. One of these models gave a possible solution, with residues 35–65 well placed in the electron density maps generated by Phaser^9^. A subsequent search model using only these residues gave a definite solution and the remaining residues other than the first 11 could be manually built into the electron density to give a final model at 1.85 Å with an R_free_ of 0.223 (Fig. 1).

### Overall structure of GapR

In this structure, there is a single GapR monomer in the asymmetric unit. It has an extended N-terminal α helix followed by two shorter α helices (Fig. 1a). The three helices are relatively open and not bundled together, suggesting that this conformation would not be stabilized in the absence of a binding partner. Indeed, applying crystallographic symmetry assembles the monomers into repeating tetramer units (Fig. 1c) which have a large central channel. The tetramer is very similar, but not identical to the published tetramer^5^.

The same crystallization condition also produced crystals with a second morphology, which were of the space group P222_1_. The diffraction from these crystals displayed severe anisotropy and had resolution limits between 2.0 and 2.7 Å. Following ellipsoidal truncation and anisotropic correction using the Diffraction Anisotropy Server^10,11^, the structure was solved using our first GapR structure as a molecular replacement search model and refined to a final R_free_ of 0.279. In this crystal, GapR is a dimer in the asymmetric unit, with the tetramer again being formed by crystallographic symmetry. The tetramer could be overlaid onto the I4_1_32 tetramer with a root-mean-square deviation (rmsd) of 1.5 Å (Fig. 1d).

We were also able to solve the structure of GapR_Δ1-11_ from crystals of space group P4_3_22. Diffraction from these crystals also showed high anisotropy, with resolution limits between 3.3 Å and 3.8 Å. Following ellipsoidal truncation and anisotropic correction using the Diffraction Anisotropy Server^10^ the structure was refined to an R_free_ of 0.322. There are six monomers in the asymmetric unit, arranged as a tetramer and a dimer. The tetramer could be overlaid onto the I4_1_32 tetramer with an rmsd of 1.9 Å (Fig. 1d). Crystallographic symmetry again showed that the dimer of the asymmetric unit paired with symmetry-related molecules to form tetramers. The main difference between GapR in the three crystal forms arises from slight changes in the orientation of helix three, which results in minor variations in the size of the central channel (Fig. 1d).

The central channel seen in the GapR tetramers is an obvious binding site for DNA. Interestingly, the channel is quite short, at around ~30 Å long. This is much shorter than the length of the 19-bp oligonucleotide, which would be ~65 Å long. In each crystal, GapR forms a complete and tight lattice (Fig. 1e–g), suggesting that although the presence of the DNA was required for crystal growth, it did not participate in crystal packing, and that there is not a 1:1 stoichiometry between 19 bp DNA and GapR tetramer. Furthermore, in the I4_1_32 and P4_3_22 structures, a 19 bp DNA oligio would have to adopt substantial bends to thread through the central channel of adjacent tetramers, because channels formed by adjacent GapR tetramers do not line up.

**Figure 2:**
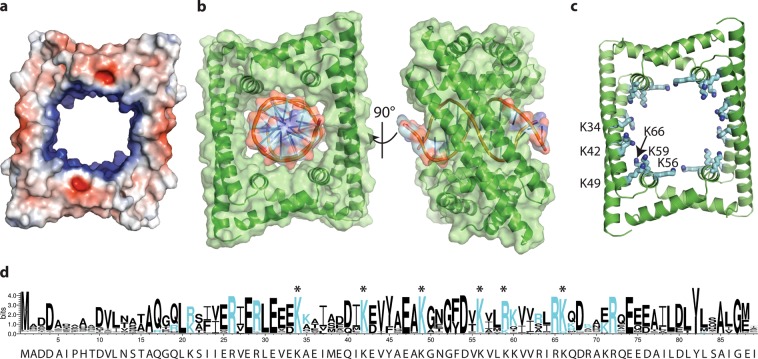
Identification and analysis of a DNA binding channel. (**a**) Electrostatic surface representation of GapR with electropositive and electronegative regions colored blue and red respectively. (**b**) The GapR tetramer (green cartoon with transparent surface representation overlaid) channel of the I4_1_32 crystal form can accommodate B-DNA (shown as sticks overlaid with transparent surface representation). B-DNA (PDBID: 1BNA)^12^ was manually placed into the GapR channel using PyMOL (The PyMOL Molecular Graphics System, Version 2.0 Schrödinger, LLC) and then subject to a round of energy-minimization in Phenix^24^. (**c**) Six highly conserved channel-lining lysine residues were identified and targeted for mutation. (**d**) Weblogo3^26^ showing sequence conservation in the 250 proteins in the NCBI protein database which share highest percentage identity with GapR from *C. crescentus* (shown beneath). The lysine residues drawn as sticks in panel C are marked with an asterisk.

An electrostatic surface representation of the GapR tetramer showed that the lining of the channel is highly positively charged (Fig. 2a). We manually placed model B-DNA (PDB ID: 1BNA)^12^ into the channel and found that it could be fit without clashing with the GapR tetramer (Fig. 2b). Closer analysis of the channel identified six highly conserved lysine residues per monomer (Fig. 2c,d) that seemed likely to contribute to DNA binding by tetrameric GapR.

### GapR is a tetramer in solution that binds DNA through conserved lysine residues

**Figure 3:**
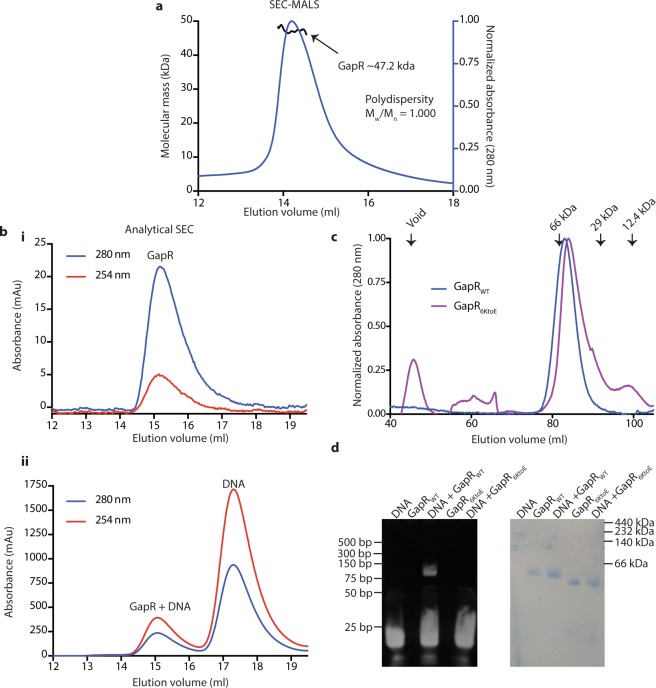
Mutation of channel lysines abolishes DNA binding but does not disrupt tetramer formation. (**a**) SEC-MALS of apo GapR. Absorbance at 280 nm (blue) and the calculated scattering mass across the peak (black) are shown. The expected mass of tetrameric GapR is 40.9 kDa. (**b**) Analytical size exclusion chromatography of GapR (6.25 μM tetramer, ε_280_ = 2980 M^−1^ cm^−1^) in the (i) absence and (ii) presence of 19 bp DNA (20 μM, ε_260_ = 309,776 M^−1^ cm^−1^) using a Superdex 200 Increase 10 300 GL column. (**c**) Overlay of size exclusion chromatograms for GapR (blue) and GapR_6KtoE_ (purple) GapR. Samples of 6.25 μM (if calculated using the tetrameric molecular weights) protein was applied to a HiLoad 16/600 Superdex 200 pg column. Arrows indicate the void volume of the column and elution positions of proteins standards of known molecular weight (66 kDa – bovine serum albumin, 29 kDa – carbonic anhydrase, 12.4 kDa – cytochrome *c*. (**d**) EMSA experiment demonstrating that DNA binding by GapR is mediated by conserved lysine residues lining the tetrameric channel. Wild type GapR or GapR_6KtoE_ (0.425 μM tetramer) was incubated in the presence or absence of 19 bp oligonucleotide (1.7 μM) and used for non-denaturing gel electrophoresis. The gel was stained with SYBR Gold to detect DNA (left side of panel), then with InstantBlue to detect protein (right side of panel). Note that GapR has an overall negative charge. Uncropped gels are presented in Supplementary Figure 1.

We used size exclusion chromatogram – multiangle light scattering (SEC-MALS) to investigate the oligomeric state of GapR in solution (Fig. 3a). SEC-MALS of GapR in the absence of DNA provided an estimate of its solution molecular weight of ~47.2 kDa, most consistent with a non-compact GapR tetramer. Analytical size exclusion chromatogram (SEC) of GapR in the presence and absence of the 19 bp DNA used for crystallization demonstrated that DNA binding does not alter the oligomeric state of GapR (Fig. 3b). Thus the analytical SEC and SEC-MALS show that GapR can exist as a tetramer in solution, even in the absence of DNA.

We next tested if the conserved lysines (Fig. 2) did contribute to DNA binding. We mutated all six of these lysines to aspartic acids, and purified the resulting protein (GapR_6KtoE_). GapR_6KtoE_ showed low solubility, which has also been reported for wild type GapR in the absence of DNA^4^, and it could not be concentrated above 0.4 mg/ml. To ensure the mutations did not disrupt tetramerization, we compared the oligomeric state of the mutant and wild type proteins by migration in size exclusion chromatography. GapR_6KtoE_ migrated slightly slower than wild type GapR (Fig. 3c), but comparison with proteins of known molecular weights show this small difference is not indicative of a change in oligomeric state. To assess DNA binding of wild type and mutant proteins, we performed an electrophoretic mobility shift assay (EMSA) (Fig. 3d). DNA binding was observed for wild type GapR, but not for GapR_6KtoE_. Like wild type protein, GapR_6KtoE_ migrated through the native gel as a single band (Fig. 3d) but slightly faster than wild type GapR. The small differences in migration is likely caused by the difference in charge introduced by the six lysine to aspartic acid mutations per monomer (24 per tetramer). The inability of GapR_6KtoE_ to bind DNA supports the conclusion that GapR binds DNA through its central pore^5^.

### The GapR channel can accommodate B-form DNA

**Figure 4:**
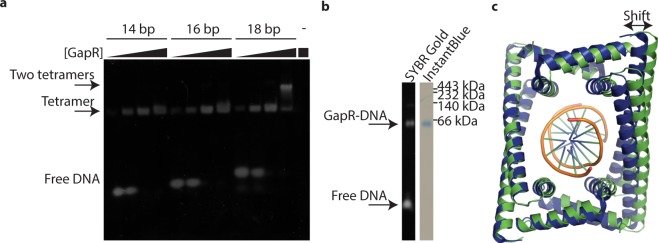
GapR DNA-binding analysis. (**a**) EMSA analysis of GapR binding to DNA oligonucleotides of different lengths. 0.4 μM DNA oligonucleotide (Table 1) was incubated with GapR (of concentrations 0.4, 1.2, 3.6 and 10.8 μM, if calculated using monomeric molecular weight or 0.1, 0.3, 0.9 and 2.7 μM if tetramer) prior to non-denaturing gel electrophoresis. GapR (2.7 μM tetramer) without oligonucleotide was analysed in the rightmost lane. The uncropped gel is presented in Supplementary Figure 1. (**b**) Crystals of GapR contain DNA. Crystals of space group I4_1_32 crystals, grown from a sample of GapR with 8 bp oligonucleotide were subject to gel electrophoresis and stained for the presence of DNA (left side of panel) and protein (right side of panel). It is likely the free DNA observed here results from disassociation of the oligo after harvesting & washing the crystal and when loading onto the gel, when it is no longer in excess. The uncropped gel is presented in Supplementary Figure 1. (**c**) Overlay of the crystal structure determined from crystals of space group I4_1_32 (green) with the GapR-DNA structure of Guo *et al*.^5^ (blue with DNA shown in orange, PDB ID: 6CG8). The Guo *et al*. structure features a marginally narrower channel.

Modelling of DNA into our GapR structures revealed that our original 19-bp oligonucleotide was not ideal for structural studies, as GapR would be able to bind at various locations along the 19-mer. To determine the optimal length of DNA required for GapR binding we performed EMSA experiments. We reasoned that when GapR is in excess over DNA, multiple copies of the GapR tetramer should bind to DNA if its length is sufficient. By using increasing lengths of oligonucleotides in EMSA experiments it should be possible to measure the transition that accommodates extra GapR binding as the threshold length is crossed. Accordingly, we performed EMSA with excess GapR and 14, 16 and 18-bp DNA oligonucleotides. GapR bound all DNA oligomers, but a second shift was observed only with the 18-bp DNA (Fig. 4a). Thus, a segment of 18 base pairs of DNA is just sufficient to bind two GapR tetramers, suggesting that a single GapR binds to ~9 base pairs of DNA.

We next repeated our crystallization protocols with GapR in the presence of DNA oligos of 8, 9 and 10 base pairs. Crystals with identical morphology to those grown with 19-bp DNA were obtained for the 8-bp DNA, but despite the inclusion of excess DNA oligonucleotide during cryoprotection, no density for DNA could be seen in the electron density maps of the resulting structure. A native gel of washed, dissolved crystals demonstrated that DNA was in fact present (Fig. 4b). Thus, the absence of DNA in the crystal structures suggests that the DNA binds in a continuum of positions within the channel. Indeed, the lysine residues are fairly evenly spread out in the channel, and a mechanism to keep the DNA at any one particular position within the channel is not evident.

## Discussion

During the course of this work, a structure of GapR in complex with DNA was published^5^. GapR forms essentially the same tetramer seen in our structures, with DNA bound in the central channel, as we anticipated. The six lysines that we identified, plus an additional arginine, all make contacts with the DNA backbone. In marked contrast to our study, which used DNA with an AT content ranging from 40 to 53%, the DNA used by Guo *et al*. was 100% AT. This DNA adopted an over-twisted conformation, with a wider major groove and narrower minor groove than seen in B-DNA. The length of this DNA oligomer is 11 base pairs and it forms head-to-tail contacts with the adjacent symmetry-related DNA molecule to form extended noncovalent DNA polymers. The length and AT content of the DNA were likely both important for crystallization and the ordering that allowed the DNA to be visualized.

Over-twisting of DNA slightly narrows its diameter relative to B-DNA and the DNA-bound GapR tetramer^5^ is constricted relative to our structure, with a narrower DNA channel (Fig. 4c). Guo *et al*. report being unable to fit B-DNA into their GapR tetramer without clashes^5^, although these appear to be minor clashes with mobile side chains. In contrast, our analysis of our structure indicates the tetramer we observe could comfortably accommodate B-DNA (Fig. 2b). In addition to crystallizing tetrameric GapR bound to DNA, Guo *et al*. were also able to crystallize GapR from the closely related *Bosea* sp. Root381, in the presence and absence of DNA. In the presence of DNA, which was visible in electron density maps but could not be reliably modelled, GapR was tetrameric. In the absence of DNA, GapR crystallized as a dimer, with α helices 2 and 3 rearranging to form a single extended helix^5^. On the basis of these structures, the authors proposed a model for DNA binding whereby dimeric GapR uses its two extended helices to track along DNA, monitoring the size of the major and minor grooves. Upon encountering over-twisted DNA, the extended α-helix re-organizes into two shorter helices and forms the stable tetramer with a co-translocating dimer. In our studies with GapR from *C. crescentus*, we saw no evidence of the dimer form in the presence or absence of DNA (Fig. 3). However, we did note some subtle difference in position of helix three in each of our crystal forms (Fig. 1d), suggesting that plasticity of this region could possibly be important for formation and size of the central channel.

**Figure 5:**
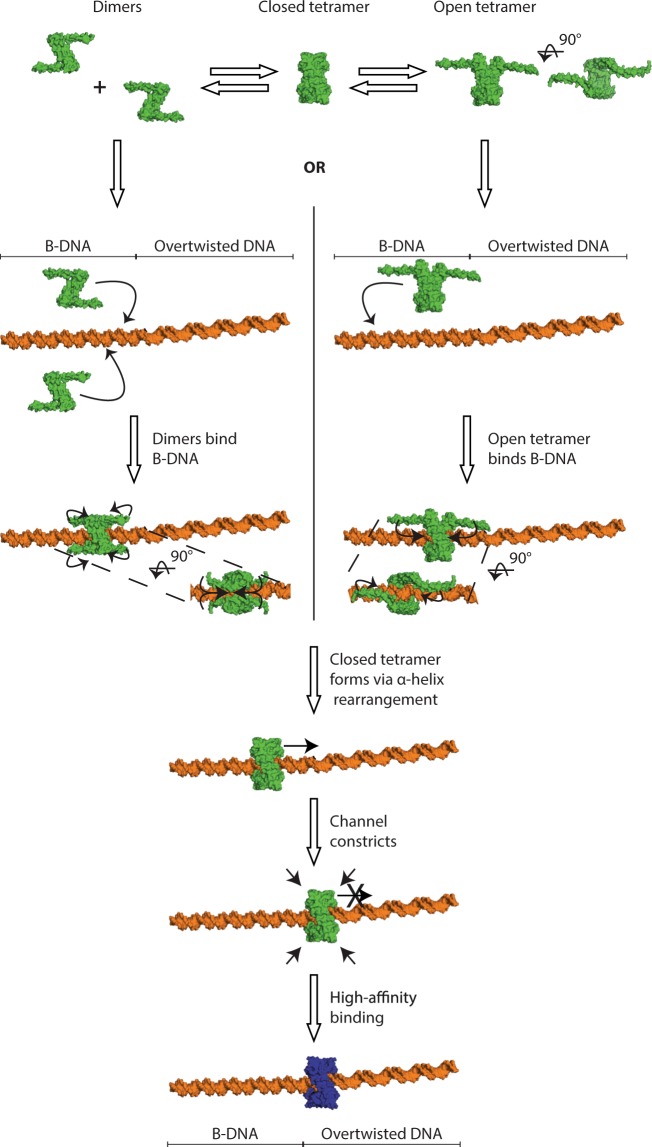
Proposed models for GapR binding to DNA. GapR first associates with DNA via one of two possible pathways. In the pathway shown to the left, GapR dimers^5^ (PDB ID: 6CFY) associate with DNA weakly until encountering a partner. The two dimers then assemble into a GapR tetramer by rearrangements in the C-terminal α helices and encirclement of the DNA. In the pathway shown on the right, a pre-formed GapR tetramer encircles DNA through rearrangement of two C termini on one side of the tetramer. If the tetramer has formed around B-form DNA, it adopts a more open conformation and can freely diffuse along the DNA. Upon encountering over-twisted DNA, GapR constricts slightly (as in PDB ID: 6CG8^5^), narrowing the channel around the DNA and leading to a higher-affinity complex.

That the current structures show GapR tetramers can accommodate B-DNA in the channel, and that we observe tetrameric GapR in absence of DNA, has important implications for the model of DNA binding (Fig. 5). GapR could first associate with B-DNA as a dimer, with the region of helices 2 and 3 extended into a single long helix, like in the Guo model, or as a tetramer, which would open to allow DNA binding by splaying of two interacting copies of helices 2 and 3. In both proposed pathways, the tetramer would close around DNA by rearrangement into the α helices 2 and 3 into the conformation observed in our structures, with the wider GapR tetramer bound to B-DNA. GapR with this wider channel would scan along B-DNA until it encounters over-twisted, AT-rich DNA. The over-twisted DNA would induce the transition to the slightly constricted tetramer, allowing it to bind the over-twisted, AT-rich DNA with higher affinity^5^, thus localizing GapR to this site.

A key point of the updated model is the scanning of DNA by the GapR tetramer. GapR encircling and scanning B-DNA could be more processive and efficient than scanning as a dimer, as it could proceed by one-dimensional diffusion rather than repeated association and disassociation. The model also allows GapR tetramers to form on B-form DNA in the bacterial genome, and not just at over-twisted DNA, which obviates the less probable event of two GapR dimers being coincidentally attracted to the same over-twisted DNA. It is also not clear how a GapR dimer would be preferentially attracted to over-twisted DNA. Scanning of B-DNA by tetrameric GapR remains to be directly shown, but passive movement along double stranded DNA has ample precedence in proteins which encircle DNA such as sliding clamps, MutS-ATP, Ku70/80 and proliferating cell nuclear antigen^13–18^. It is also possible that interactions with other proteins in the cell could affect the size of the channel and the sliding rate along the DNA.

The GapR binding and scanning model also help explain previous observations that seemed significant for biological function yet lacked a mechanistic basis. Having GapR tetramers sliding across a predominantly CG-rich genome and occasionally encountering AT-rich patches where presumably they pause with a tighter conformation and a narrower channel could help reconcile the differential reporting of a strong^2^ or weak preference for AT-rich DNA *in vivo*^3–5^. It is possible that the FLAG-tagged GapR used by Ricci *et al*. somehow favored the tighter conformation, accentuating the preference for AT-rich DNA.

More importantly, the model has implications before and during chromosome replication. The GapR gradient in pre-replication *C. crescentus* “swarmer” cells is easier to explain if GapR tetramers can bind relaxed DNA. Also, *C. crescentus* GapR is required at the start of chromosome replication and during the first stage of chromosome separation that coincides with early DNA duplication^3^. ChIP experiments in synchronized cells showed distinct high and low patterns of GapR binding at each of the “left”, “middle” and “right” positions within a short (~700 bp) span of the *C. crescentus* chromosome origin of replication. Such localized and DNA-specific binding patterns probably result from GapR encounters with DNA sequence-specific replication proteins as well as RNA and DNA polymerases. GapR binding and sliding would be important for reaching targets inside the origin of replication and similarly important to minimize interference with essential replication proteins on a crowded DNA platform^19^.

## Materials and Methods

### Oligonucleotides

Single stranded DNA oligonucleotides used in this study are listed in Table 1. To anneal them into double stranded DNA, oligonucleotides were resuspended in annealing buffer (10 mM Tris pH 7.5, 50 mM NaCl, 1 mM EDTA) at 100 μM. Complementary strands were mixed at a 1:1 ratio, incubated at 95 °C for five minutes and then slow-cooled to room temperature.

**Table 1:** DNA oligonucleotides used in this study.

Name	Sequence (5′ to 3′)	Source
GapR_Fwd	AAAACCATGGCCGACGACGCCATTCC	Integrated DNA Technologies
GapR_Rev	AAAAGAATTCAACGCTCGACCATACGTCTC	Integrated DNA Technologies
GapR_NtDel_Fwd	ACCTGTATTTTCAGGGCCTGAACTCGACCGC	BioCorp DNA
GapR_NtDel_Rev	GCGGTCGAGTTCAGGCCCTGAAAATACAGGT	BioCorp DNA
19mer_Fwd	GTTAAGCAACCGTTAACGG	BioCorp DNA
19mer_Rev	CCGTTAACGGTTGCTTAAC	BioCorp DNA
10mer	CCGTTAACGG	Integrated DNA Technologies
9mer_Fwd	CCGTTAACG	Integrated DNA Technologies
9mer_Rev	CGTTAACGG	Integrated DNA Technologies
8mer	CGTTAACG	Integrated DNA Technologies

**Table 2:** Data collection and refinement statistics.

	6OZX	6OZY		6OZZ	
**Data collection**					
Wavelength (Å)	0.979	0.979		0.979	
Resolution range (Å)	82.36–1.85 (1.89–1.85)	50.0–2.00 (2.05–2.00)		50.0–3.30 (3.38–3.30)	
Space group	I4_1_32	P222_1_		P4_3_22	
Unit cell *a b c (Å) α β γ (°)*	116.5 116.5 116.5 90 90 90	39.7 59.3 93.9 90 90 90		106.3 106.3 139.1 90 90 90	
		Pre-correction	Post-correction	Pre-correction	Post-correction
Total reflections	821532 (38807)	201701 (13944)	130424 (589)	323588 (22821)	242400 (1083)
Unique reflections	11872 (709)	15659 (1134)	10203 (50)	12618 (901)	9555 (49)
Multiplicity	69.2 (54.7)	12.9 (12.2)	8.5 (0.5)	25.6 (25.3)	19.3 (1.2)
Completeness (%)	99.9 (98.2)	99.9 (99.3)	66.5 (4.5)	99.9 (99.8)	76.1 (5.4)
*Mean I/σ(I)*	30.2 (2.6)	10.9 (0.5)	16.5 (3.1)	13.8 (0.5)	18.0 (3.1)
*R*_*merge*_	0.113 (2.45)	0.137 (4.74)	0.095 (0.808)	0.156 (7.88)	0.116 (1.25)
*CC*_*1/2*_	0.999 (0.532)	0.999 (0.349)	0.998 (0.809)	0.998 (0.367)	0.999 (0.880)
**Refinement**					
Resolution range (Å)	47.55–1.85 (1.917–1.85)	46.97–2.01 (2.09–2.01)		49.7–3.30 (3.42–3.30)	
*R*_*work*_	0.193 (0.255)	0.2373 (0.356)		0.2852 (0.409)	
*R*_*free*_	0.223 (0.282)	0.279 (0.357)		0.322 (0.461)	
Non-hydrogen atoms	673	1342		3661	
Macromolecules	627	1251		3661	
ligands	—	1		—	
water	46	90		0	
Protein residues	78	156		455	
RMS bonds (Å)	0.011	0.002		0.003	
RMS angles (°)	1.21	0.42		0.58	
Ramachandran favored (%)	100	100		100	
Clashscore	0.00	0.00		2.39	
Average *B*-factor	41.6	49.6		146	
macromolecules	41.2	49.7		146	
ligands	—	45.7		—	
solvent	46.6	48.0		—	

Statistics for the highest-resolution shell are shown in parentheses.

### Cloning, expression and purification of GapR constructs

Full length GapR was amplified from plasmid pJT160^3^ using primers GapR_Fwd and GapR_Rev (Table 1). The PCR product was digested with *Nco*I and *Eco*RI (New England Biosciences) and ligated into a similarly digested pJ411-derived vector containing an N-terminal TEV cleavable octa-histidine tag. GapR_Δ1-11_ was generated by site-directed mutagenesis by deleting the first eleven codons of *gap*R with primers GapRNtDelF and GapRNtDelR. GapR_6KtoE_ was synthesised by ATUM (Newark, California) and cloned into pUC57 between the *Nco*I and *Not*I restriction sites. This region was then subcloned into the *Nco*I/*Not*I digested pJ411-derived vector described above.

Expression of GapR, GapR_Δ1-11_ and GapR_6KtoE_ was induced in *Escherichia* coli ΒL21(DE3) cells grown at 30 °C in LB media to an OD_600_ of ~0.6, before inducing with 1 mM isopropyl β-D-1-thiogalactopyranoside. Cell cultures were grown for five more hours at 30° before harvesting. Cell pellets were stored at −80 °C until required.

Pellets were resuspended in IMAC binding buffer (20 mM HEPES pH 7.4, 200 mM NaCl, 40 mM imidazole, 10% glycerol, 2 mM β-mercaptoethanol (βME)) supplemented with 1 mM PMSF and several crystals of DNase I. Cells were lysed by sonication (5 minutes total pulse at 50% amplitude, 10 s on, 20 s off) and the lysate cleared by centrifugation at 18 000 *g*. Cleared lysates were applied to a 5 ml HisTrap IMAC FF column (GE Healthcare) and washed with heparin elution buffer (20 mM HEPES pH 7.4, 2 M NaCl, 10% glycerol, 2 mM βME). After re-equilibration with IMAC binding buffer, bound protein was eluted with IMAC elution buffer (as binding buffer but 800 mM imidazole). Fractions containing GapR were pooled and applied to a 5 ml HiTrap Heparin HP column (GE Healthcare) and bound protein eluted on a 100 ml gradient to 60% heparin elution buffer. Protein-containing fractions (GapR_6KtoE_ failed to bind the column and protein was recovered from the flow through) were pooled and the His tag cleaved during overnight dialysis against 1 L of IMAC binding buffer in the presence of TEV protease^20^ at 4 °C. Samples were reapplied to the HisTrap column and the flow-through collected. For formation of GapR-DNA and GapR_Δ1-11_-DNA complexes for crystallization, excess annealed oligonucleotides (Table 1) were added and the resultant complex purified on a HiLoad 16/600 Superdex 200 pg column (GE Healthcare) in SEC buffer (20 mM HEPES pH 7.4, 200 mM NaCl, 0.2 mM TCEP) to remove unbound DNA.

### Crystallography

GapR-DNA and GapR_Δ1-11_-DNA complexes were concentrated to 3 mg/ml, as determined by the Bio-Rad Protein Assay, (Bio-Rad Laboratories) using 3 kDa MWCO Amicon Ultra centrifugation devices (EMD Millipore) and subjected to sparse-matrix crystallization against commercially available screens (Qiagen) using sitting drop vapor diffusion at room temperature. Crystals were obtained in multiple conditions, with final conditions optimized in 24-well sitting drop plates with 500 μl reservoir and 2 μl protein sample plus 2 μl reservoir solution in the drop. The final crystallization conditions for the I4_1_32 and P222_1_ crystal forms of GapR-DNA were 0.16 M ammonium sulfate, 12% PEG 3350 with 1% 1,2-butanediol or 10 mM cadmium chloride respectively. Crystals with morphologies indicative of both crystal forms were seen in both conditions. GapR_Δ1-11_-DNA crystallized in space group P4_3_22 in 4% PEG 3350, 0.16 M ammonium sulfate, 0.6% 1,2-butanediol. Crystals were transferred to the reservoir solution used for crystallization that additionally contained all the components of SEC buffer and 20–30% glycerol and then cryo cooled in liquid nitrogen. Datasets were collected on beamline 08ID-1 of the CMCF at the Canadian Light Source, using light of 0.979 Å wavelength in Saskatoon, SK, Canada.

Datasets were indexed with the program iMosflm^21^ and scaled with the program AIMLES^22^. The P222_1_ and P4_3_22 datasets displayed high levels of anisotropy and were re-indexed and re-scaled in XDS^23^ prior to submission to the diffraction anisotropy server^10,11^. Ellipsoidal resolution boundaries of 2.6, 2.0 and 2.3 Å for the P222_1_ dataset and 3.8, 3.8 and 3.3 Å for the P4_3_22 dataset were applied along the a*, b* and c* axes respectively and anisotropically scaled using the webserver. Data collection statistics for the pre- and post-anisotropically corrected datasets are presented in Table 1.

Structure determination of GapR in the I4_1_32 space group was performed by molecular replacement in the Phaser module of PHENIX^24^ using models generated by the Roβetta server^8^ from the GapR sequence. This produced a partial solution for residues 35–66. After using these residues alone as a molecular replacement model, clear density for the remaining residues was visible in the resulting maps. GapR was manually built into the maps in the program Coot^25^ followed by refinement in PHENIX to produce the final model (Table 1). This structure was then used as a search model to determine the structure of GapR in space groups P222_1_ and P4_3_22 by molecular replacement using Phaser followed by iterative rounds of model building and refinement in Coot and PHENIX. Figures were generated using PyMOL (The PyMOL Molecular Graphics System, Version 2.0 Schrödinger, LLC).

### SEC-MALS

A sample of 50 μl of GapR at 0.5 mM concentration (when calculated using tetrameric molecular weight) was applied to a Superdex-200 Increase 10 300 GL column (GE Healthcare) equilibrated in SEC buffer, attached to in-line miniDAWN TREOS (Wyatt Technologies) and Optilab rEX (Wyatt Technologies) SEC-MALS instruments. Molecular mass from SEC-MALS data was calculated with the ASTRA 5.3.4.20 (Wyatt Technologies) software. Lower concentrations of GapR eluted at the same volume but gave noisier scattering. Bovine serum albumin was used as a standard.

### Gel electrophoresis

For the EMSA experiments, GapR was incubated with DNA in annealing buffer and subjected to electrophoresis in an 8%, 0.5 X TBE gel at 100 V for 60–80 minutes in 0.5 X TBE buffer pH 8.3 at 4 °C. DNA was visualized with SYBR Gold (Thermo Fisher Scientific) and imaged with an AlphaDigiDoc gel documentation system (Alpha Innotech). Protein complexes were stained with InstantBlue (Sigma).

For gel electrophoresis analysis of GapR-DNA crystals, 10 crystals were each looped into 1 μl stabilization buffer (20 mM HEPES pH 7.4, 200 mM NaCl, 0.2 mM TCEP, 4% PEG 3350, 0.16 M ammonium sulfate, 1% 1,2 butanediol), then looped into 20 μl of annealing buffer prior to electrophoresis as described above for EMSA.

## Supplementary information

Supplementary Information

## Data Availability

GapR structures determined in this study are available from the Protein Data Base (PDB) under the accession codes 6OZX, 6OZY and 6OZZ.
