# Supplementary material for: Structures of GapR reveal a central channel which could accommodate B-DNA
Source: Sci Rep. 2019 Nov 13;9:16679. doi: 10.1038/s41598-019-52964-2 (PMC6853979; doi:10.1038/s41598-019-52964-2)
Supplement: Supplementary file 1 — Supplementary Information [file 41598_2019_52964_MOESM1_ESM.pdf]

Structures of GapR reveal a central channel which could accommodate B-DNA

Michael J. Tarry, Christoph Harmel, James A. Taylor, Gregory T. Marczynski and T. Martin Schmeing

Supplementary Information

Figure S1

Original gel images prior to being cropped for presentation in main article figures

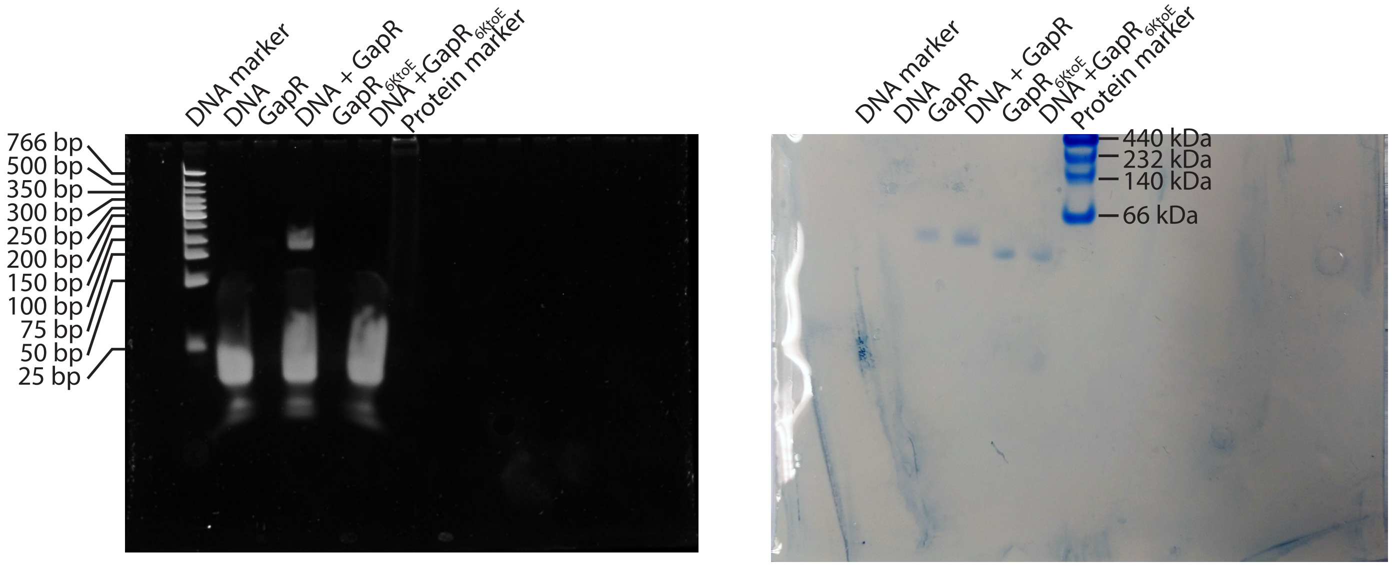

Original gels related to Figure 3a

Original gel images after staining with Sybergold (left) and InstantBlue (right) used to generate the cropped panels in Figure 3a

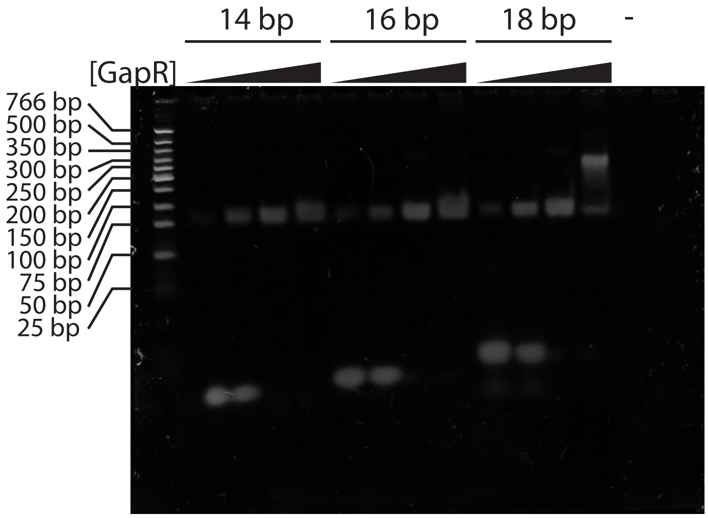

Original gels related to Figure 4a

Original gel image after staining with Sybergold used to generate the cropped panel in Figure 4a

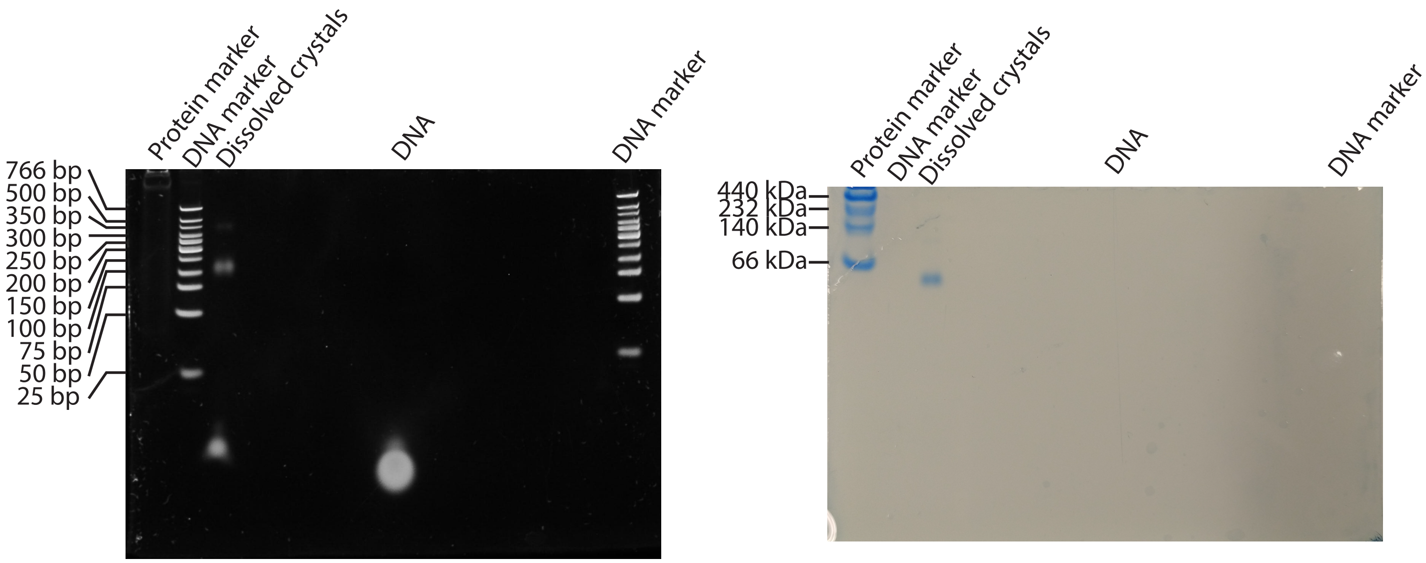

Original gels related to Figure 4b

Original gel image after staining with Sybergold (left) and InstantBlue (right) used to generate the cropped panels in Figure 4b
